# CCAR‐1 is a negative regulator of the heat‐shock response in *Caenorhabditis elegans*

**DOI:** 10.1111/acel.12813

**Published:** 2018-07-12

**Authors:** Jessica Brunquell, Rachel Raynes, Philip Bowers, Stephanie Morris, Alana Snyder, Doreen Lugano, Andrew Deonarine, Sandy D. Westerheide

**Affiliations:** ^1^ Cell Biology, Microbiology and Molecular Biology University of South Florida Tampa Florida USA

**Keywords:** *C. elegans*, CCAR‐1, heat‐shock response, HSF‐1, longevity, SIR‐2.1

## Abstract

Defects in protein quality control during aging are central to many human diseases, and strategies are needed to better understand mechanisms of controlling the quality of the proteome. The heat‐shock response (HSR) is a conserved survival mechanism mediated by the transcription factor HSF1 which functions to maintain proteostasis. In mammalian cells, HSF1 is regulated by a variety of factors including the prolongevity factor SIRT1. SIRT1 promotes the DNA‐bound state of HSF1 through deacetylation of the DNA‐binding domain of HSF1, thereby enhancing the HSR. SIRT1 is also regulated by various factors, including negative regulation by the cell‐cycle and apoptosis regulator CCAR2. CCAR2 negatively regulates the HSR, possibly through its inhibitory interaction with SIRT1. We were interested in studying conservation of the SIRT1/CCAR2 regulatory interaction in *Caenorhabditis elegans*, and in utilizing this model organism to observe the effects of modulating sirtuin activity on the HSR, longevity, and proteostasis. The HSR is highly conserved in *C. elegans* and is mediated by the HSF1 homolog, HSF‐1. We have uncovered that negative regulation of the HSR by CCAR2 is conserved in *C. elegans* and is mediated by the CCAR2 ortholog, CCAR‐1. This negative regulation requires the SIRT1 homolog SIR‐2.1. In addition, knockdown of CCAR‐1 via *ccar‐1* RNAi works through SIR‐2.1 to enhance stress resistance, motility, longevity, and proteostasis. This work therefore highlights the benefits of enhancing sirtuin activity to promote the HSR at the level of the whole organism.

## 1 INTRODUCTION

Maintaining the quality of the proteome is essential for cellular homeostasis, and an accumulation of misfolded proteins is a feature of many aging‐related diseases (Labbadia & Morimoto, 2015). A conserved mechanism to maintain proteostasis is through induction of the cytoprotective heat‐shock response (HSR), regulated by the transcription factor heat‐shock factor 1 (HSF1) (Gomez‐Pastor, Burchfiel, & Thiele, 2017). HSF1 functions to protect cells from protein‐damaging stress through the transcription of heat‐inducible heat‐shock protein (*hsp*) genes, which encode protein chaperones that assist in protein folding and clearance (Kim, Hipp, Bracher, Hayer‐Hartl, & Hartl, 2013). Increasing HSF1 activity and chaperone expression is not only beneficial during stress, but can also prevent toxic aggregate species in protein aggregation diseases (Neef, Jaeger, & Thiele, 2011). Uncovering HSR inducers to promote chaperone production is thus an active area of research.

One factor known to modulate the HSR is the sirtuin family member SIRT1 (Liu et al., 2014; Raychaudhuri et al., 2014; Raynes et al., 2013; Westerheide, Anckar, Stevens, Sistonen, & Morimoto, 2009; Zelin & Freeman, 2015). SIRT1 is part of a family of conserved NAD^+^‐dependent deacetylases and has broad roles in physiology and longevity (Haigis & Sinclair, 2010). Increased expression of SIRT1 enhances the HSR through deacetylation of the DNA‐binding domain of HSF1, thereby prolonging its DNA‐binding competent state and allowing for increased transcription of *hsp70* (Westerheide et al., 2009). SIRT1 is also essential for maintaining the proteome, as a SIRT1 deficiency results in defective protein quality control (Tomita et al., 2015). Enhancing SIRT1 activity may therefore be one strategy for promoting proteostasis.

SIRT1 activity is controlled by a number of factors. Active regulator of SIRT1 (AROS) is a positive regulator of SIRT1 that promotes deacetylation of the SIRT1 substrates p53 and HSF1 (Kim, Kho, Kang, & Um, 2007; Raynes et al., 2013). CCAR2, also known as DBC1, is a negative regulator of SIRT1 that enhances acetylation of p53 and HSF1 (Kim, Chen, & Lou, 2008; Raynes et al., 2013; Zhao et al., 2008). The ability of AROS and CCAR2 to modulate SIRT1 activity, and thus impact the acetylated state of HSF1, allows these proteins to regulate the HSR (Raynes et al., 2013). AROS enhances the HSR by promoting HSF1 binding to the *hsp70* promoter and enhancing *hsp70* mRNA expression, whereas CCAR2 dampens the HSR by decreasing HSF1 binding to the *hsp70* promoter and inhibiting *hsp70* mRNA expression (Raynes et al., 2013). Thus, sirtuin modulators impact the mammalian HSR.

*Caenorhabditis elegans* is an ideal model organism for studying the impact of genetics on physiology and longevity. The HSR is highly conserved, and *C. elegans* HSF‐1 is associated with aging and longevity (Hsu, Murphy, & Kenyon, 2003; Morley & Morimoto, 2004; Morton & Lamitina, 2013). SIRT1‐regulated processes are also conserved in the worm and are mediated by SIR‐2.1. Worms expressing extra copies of *sir‐2.1* exhibit increased longevity (Burnett et al., 2011; Rizki et al., 2011; Tissenbaum & Guarente, 2001; Viswanathan & Guarente, 2011). Also, enhancing *sir‐2.1* activity through caloric restriction enhances the transcription of *hsp‐70* (Raynes, Leckey, Nguyen, & Westerheide, 2012). Although *C. elegans* does not possess a known ortholog to AROS, CCAR2 does have a worm ortholog named CCAR‐1, previously called LST‐3 (Brunquell, Yuan, Erwin, Westerheide, & Xue, 2014). We were therefore interested in determining whether negative regulation of the HSR by the SIRT1 modulator CCAR2 also occurs in the worm, and how this interaction would impact stress‐related responses and longevity.

In this study, we have identified CCAR‐1 as a negative regulator of the HSR in *C. elegans* in a SIR‐2.1‐dependent manner. CCAR‐1 negatively regulates the HSR by modulating *hsp‐70* promoter activity, HSF‐1 acetylation, and HSF‐1 binding to the *hsp‐70* promoter during HS. A family of HS‐inducible *hsp‐70* genes is enhanced during heat shock (HS) in response to *ccar‐1* RNAi, and this effect is dependent on the deacetylase activity of SIR‐2.1. We have also found that modulating SIR‐2.1 activity via *ccar‐1* RNAi promotes stress resistance, motility, longevity, and proteostasis. This work thus supports the use of sirtuin modulators to improve proteostasis and promote healthy aging.

## 2 RESULTS

### 2.1 CCAR‐1 is a negative regulator of the *C. elegans* HSR

**Figure 1:**
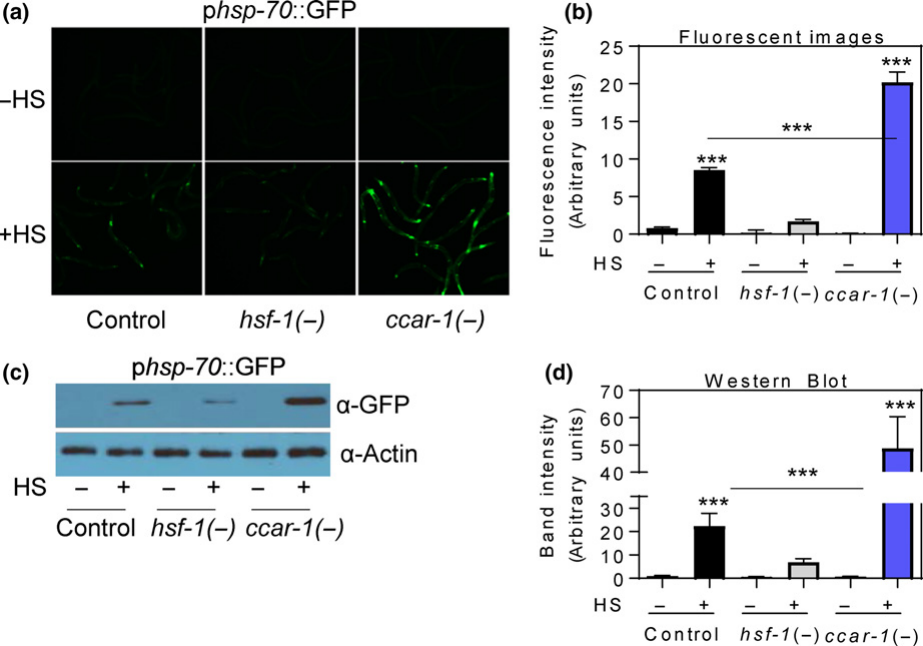
*ccar‐1* RNAi enhances *hsp‐70* promoter activity upon HS. (a) Fluorescent images are shown of *pC12C8.1*(*hsp‐70*)::GFP worms fed control RNAi, *hsf‐1* RNAi, or *ccar‐1* RNAi from the L1 larval stage to the L4 larval stage prior to treatment with or without a 15‐min 33°C HS, followed by a 6‐hr recovery. Three independent biological repeats were performed for the images, and representative images are shown. (b) Quantification of GFP intensity for 50 worms/condition for each treatment condition in (a) was determined using ImageJ. The average results for three biological repeats are shown. (c) GFP protein levels in worms given the same treatment conditions in (a) were determined via immunoblotting. Three independent biological repeats were performed, and a representative blot is shown. (d) Quantification of the average band intensities for the GFP immunoblots was done using ImageJ software and graphed as intensity in arbitrary units. For (b) and (d), significance was determined using the Bonferroni post hoc test, where *** *p* < 0.001

To determine whether CCAR‐1 negatively regulates the HSR in *C. elegans*, we first tested for effects on transcription driven by the *hsp‐70* promoter in response to *ccar‐1* RNAi (Figure 1). We used a *C. elegans* reporter worm strain containing the promoter of *hsp‐70* (*C12C8.1*) fused to GFP (p*hsp‐70*::GFP). This strain was fed control RNAi, *hsf‐1* RNAi, or *ccar‐1* RNAi from the L1 larval stage to the L4 larval stage prior to treatment with or without a 15‐min HS, followed by a 6‐hr recovery. Our RNAi feeding strategy is effective in decreasing HSF‐1::GFP levels by about 80% (Brunquell, Morris, Lu, Cheng, & Westerheide, 2016) and in an almost complete elimination of *ccar‐1* mRNA (Supporting Information Figure S1). HS treatment of control RNAi worms resulted in increased GFP expression as compared to the untreated control, and this was dependent on HSF‐1, as expected (Figure 1). Interestingly, *ccar‐1* RNAi enhanced GFP expression during HS as compared to the HS control. In the RNAi control, HS resulted in a ninefold increase in fluorescence intensity, which was dependent on HSF‐1. When HS was combined with *ccar‐1* RNAi, the fluorescence intensity increased by 20‐fold. We also analyzed the GFP expression of our reporter worm via immunoblot (Figure 1c), followed by quantification of band intensity by ImageJ (Figure 1d), and a similar trend was observed. Treatment with HS induced GFP expression by 25‐fold, and HS combined with *ccar‐1* RNAi resulted in a 50‐fold increase in GFP expression. Thus, *ccar‐1* RNAi increases HS‐inducible *hsp‐70* promoter activity by a magnitude of about twofold, indicating that CCAR‐1 would normally negatively regulate the HSR.

### 2.2 CCAR‐1 affects HSF‐1 acetylation and HSF‐1 binding to the *hsp‐70* promoter

**Figure 2:**
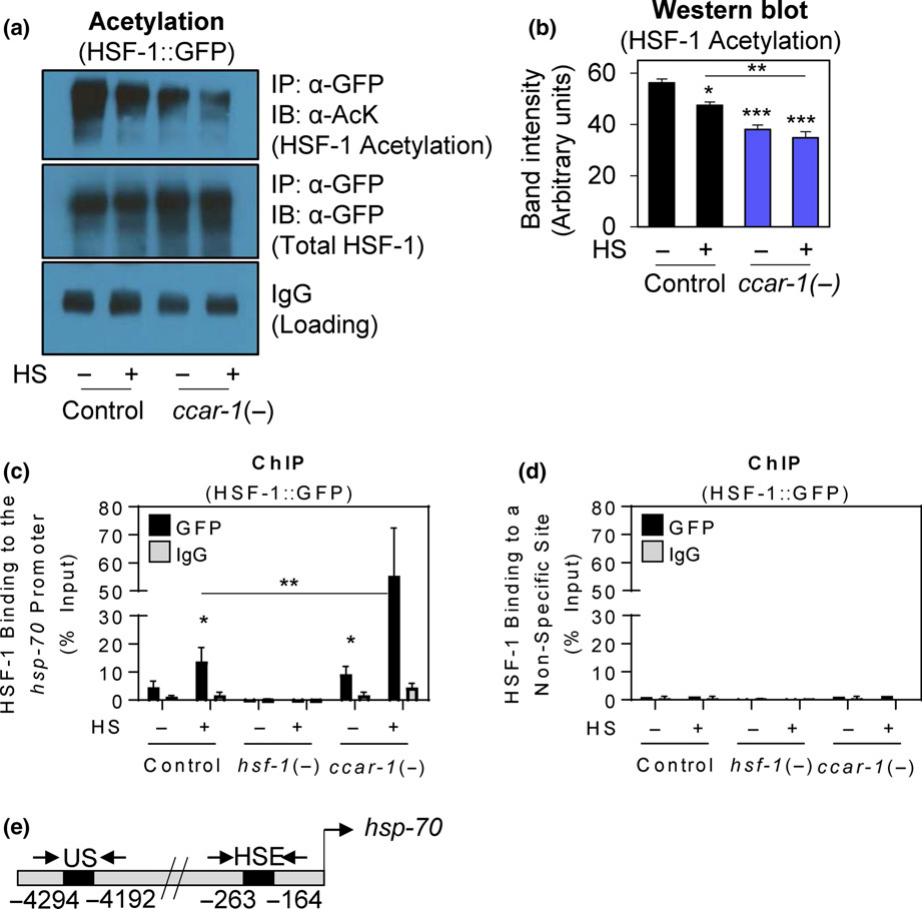
*ccar‐1* RNAi decreases HSF‐1 acetylation and increases HSF‐1 recruitment to the *hsp‐70* promoter. (a) Acetylation was assessed in HSF‐1::GFP (EQ73) worms that were fed control or *ccar‐1* RNAi from the L1 larval stage to the L4 larval stage prior to treatment with or without a 15‐min 33°C HS. HSF‐1 was then immediately immunoprecipitated (IP) using an α‐GFP antibody, and acetylation was measured by immunoblotting (IB) with an α‐AcK (acetylated lysine) antibody. Total HSF‐1 levels were measured by probing with an α‐GFP antibody. IgG bands are also shown as a loading control. (b) Quantification of band intensity for the top panel in (a) was done using ImageJ software and graphed as intensity in arbitrary units. (c) HSF‐1 binding to the *hsp‐70* promoter was assessed by performing chromatin immunoprecipitation in HSF‐1::GFP (EQ73) worms using the same treatment conditions in (a). An α‐GFP antibody was used to immunoprecipitate HSF‐1. Binding was assessed via qRT–PCR using primers designed to encompass HS elements (HSE) in the promoter region of the *C12C8.1* (*hsp‐70*) gene. (d) HSF‐1 binding to an upstream site (US) ~4 kb upstream of the *hsp‐70* promoter was assessed by performing chromatin immunoprecipitation using the same conditions as in (c). For (b) and (c), significance was determined using the Bonferroni post hoc test, where *p<0.05, **p < 0.01, ***p < 0.001. (e) A diagram of the locations of the primers used is shown, including the primers surrounding the *hsp‐70* heat‐shock element (HSE) and primers to a nonspecific (NS) upstream site

We next assessed the effects of *ccar‐1* RNAi on HSF‐1 acetylation and DNA binding to the *hsp‐70* promoter (Figure 2). The EQ73 worm strain expressing HSF‐1 tagged with GFP under the control of its own endogenous promoter (HSF‐1::GFP) (Chiang, Ching, Lee, Mousigian, & Hsu, 2012) was fed either control RNAi or *ccar‐1* RNAi from the L1 larval stage to the L4 larval stage prior to treatment with or without a 15‐min HS. To assess HSF‐1 acetylation, immunoprecipitation of HSF‐1 using an α‐GFP antibody, followed by immunoblotting with an α‐acetylated lysine antibody, was performed (Figure 2a). To quantify the total acetylation levels of HSF‐1, ImageJ was used to determine the band intensity of the α‐acetylated lysine immunoblot (Figure 2b). *ccar‐1* RNAi decreased HSF‐1 acetylation as compared to the RNAi control, both with and without HS, suggesting that CCAR‐1 would normally enhance this modification.

To measure HSF‐1 binding to the *hsp‐70* promoter, we next performed chromatin immunoprecipitation in HSF‐1::GFP worms fed control RNAi, *hsf‐1* RNAi, or *ccar‐1* RNAi from the L1 larval stage to the L4 larval stage prior to treatment with or without a 15‐min HS (Figure 2c,d). We designed ChIP primers to encompass a known HSF‐1 binding site ~200‐bp upstream of the transcription start site in the promoter of the *hsp‐70* gene *C12C8.1*, or to a sequence ~4,300‐bp upstream as a control (Figure 2e). As expected, HS increased HSF‐1 binding to the *hsp‐70* promoter 10‐fold, and this binding was abolished upon treatment with *hsf‐1* RNAi. Interestingly, *ccar‐1* RNAi increased HSF‐1 binding fivefold in the absence of HS, and 40‐fold during HS, as compared to the respective controls. We therefore conclude that CCAR‐1 would normally decrease HSF‐1 binding to the *hsp‐70* promoter.

### 2.3 CCAR‐1 regulates *hsp‐70* mRNA expression upon HS in a SIR‐2.1‐dependent manner

**Figure 3:**
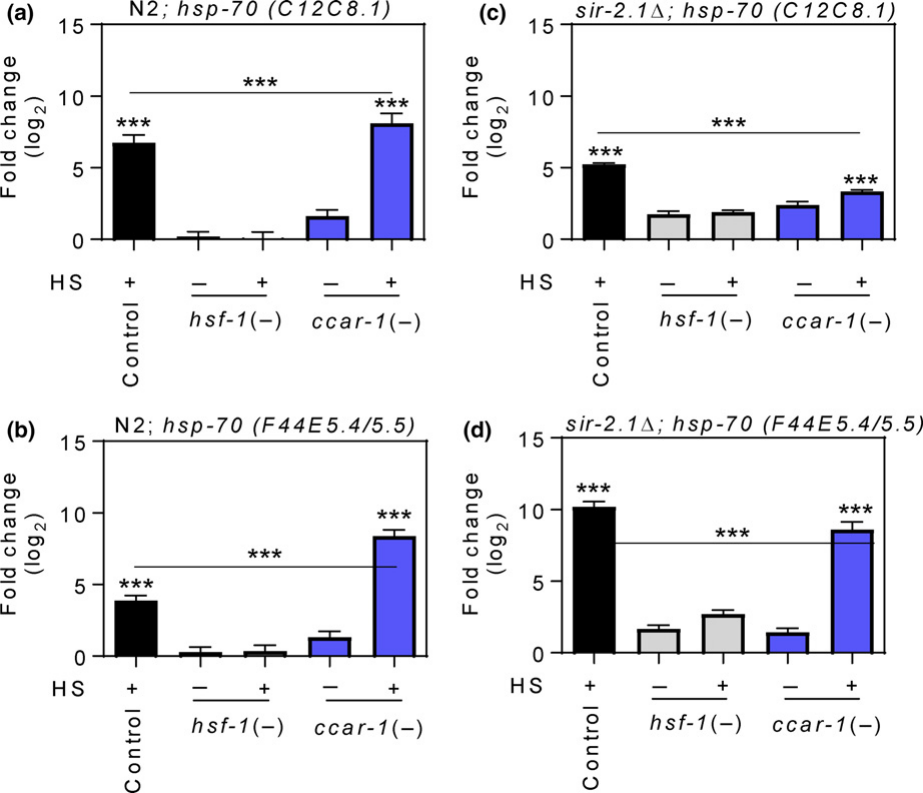
*ccar‐1* RNAi enhances a family of *hsp‐70* mRNAs in a *sir‐2.1*‐dependent manner upon HS. (a, b) qRT–PCR was used to measure the expression of the *hsp‐70* family members *C12C8.1* and *F44E5.5*/*F44E5.4* in synchronous wild‐type (N2) worms fed control RNAi, *hsf‐1* RNAi, or *ccar‐1* RNAi from the L1 larval stage to the L4 larval stage prior to treatment with or without a 15‐min 33°C HS followed by a 15‐min recovery. (c–d) qRT–PCR was used to measure the expression of the *hsp‐70* family members *C12C8.1* and *F44E5.5*/*F44E5.4* in a *sir‐2.1Δ* strain (LG339) given the same treatment conditions in (a–c). For (a–d), significance was determined using the Bonferroni post hoc test, where *p < 0.05, **p < 0.01, ***p < 0.001

To determine whether negative regulation of the HSR by CCAR‐1 in *C. elegans* is mediated through SIR‐2.1, we used qRT–PCR to measure the expression of the HS‐inducible *hsp‐70* family members *C12C8.1 and F44E5.4*/*F44E5.5* in wild‐type (N2) worms, and in LG339 worms containing a nonfunctional SIR‐2.1 protein (*sir‐2.1Δ*), in response to *ccar‐1* RNAi (Figure 3). Synchronous N2 or *sir‐2.1Δ* worms were fed control RNAi, *hsf‐1* RNAi, or *ccar‐1* RNAi from the L1 larval stage to the L4 larval stage prior to treatment with or without a 15‐min HS, followed by a 15‐min recovery. In wild‐type worms, HS treatment of the control increased the expression of both *hsp‐70* family members in an HSF‐1‐dependent manner, as expected (Figure 3a‐b). Consistent with the results in Figure 1, *ccar‐1* RNAi enhanced HS‐induced *C12C8.1* mRNA expression as compared to the HS‐treated control (Figure 3a). A similar trend is also observed for the *hsp‐70* genes *F44E5.5*/*F44E5.4* (Figure 3b). Interestingly, the ability of *ccar‐1* RNAi to enhance HS‐induced *hsp‐70* mRNA expression was abolished in the *sir‐2.1Δ* strain (Figure 3c‐d). To ensure that these results were specific to SIR‐2.1, we also examined SIR‐2.3 (a homolog to mammalian SIRT4) by performing the same analyses in the *sir‐2.3Δ* strain RB654 (Supporting Information Figure S2a–b). The ability of *ccar‐1* RNAi to enhance *hsp‐70* mRNA expression during HS is unaffected in this *sir‐2.3Δ* strain. For reasons that are not clear, the *sir‐2.1Δ* strain does not have attenuated levels of HS‐induction of C12C8.1 expression and has a higher HS‐induced fold change for the *F44E5.4/F44E5.5* genes than does the N2 strain, indicating that SIR‐2.1 may play a complex role in the regulation of chaperone genes. Nonetheless, for the *hsp* genes tested, we conclude that the enhancement of HS‐induced *hsp* mRNA levels caused by depletion of *ccar‐1* is lost upon *sir‐2.1* deletion.

We were next interested in determining whether the deacetylase activity of SIR‐2.1 was required for CCAR‐1 to negatively regulate the HSR (Supporting Information Figure S3). We used a small‐molecule selective inhibitor of mammalian SIRT1 deacetylase activity, EX‐527, which has also been shown to inhibit SIR‐2.1 in *C. elegans* (Cascella et al., 2014). First, we assessed *hsp‐70* promoter activity by feeding p*hsp‐70*::GFP worms control RNAi or *ccar‐1* RNAi, with or without EX‐527, from the L1 larval stage to the L4 larval stage prior to treatment with or without a 15‐min HS followed by a 6‐hr recovery (Supporting Information Figure S3a). Similar to the results in Figure 1, *ccar‐1* RNAi resulted in enhanced *hsp‐70* promoter activity during HS as compared to the HS control. Blocking the deacetylase activity of SIR‐2.1 by treatment with EX‐527 prevented *ccar‐1* RNAi from enhancing *hsp‐70* promoter activity during HS. This result was confirmed using qRT–PCR for the *hsp‐70* genes *C12C8.1* and *F44E5.5*/*F44E5.4* (Supporting Information Figure S3b,c). These data suggest that negative regulation of the HSR by CCAR‐1 is mediated through the deacetylase activity of SIR‐2.1.

### 2.4 *ccar‐1* RNAi promotes stress resistance and motility in a SIR‐2.1‐dependent manner

**Figure 4:**
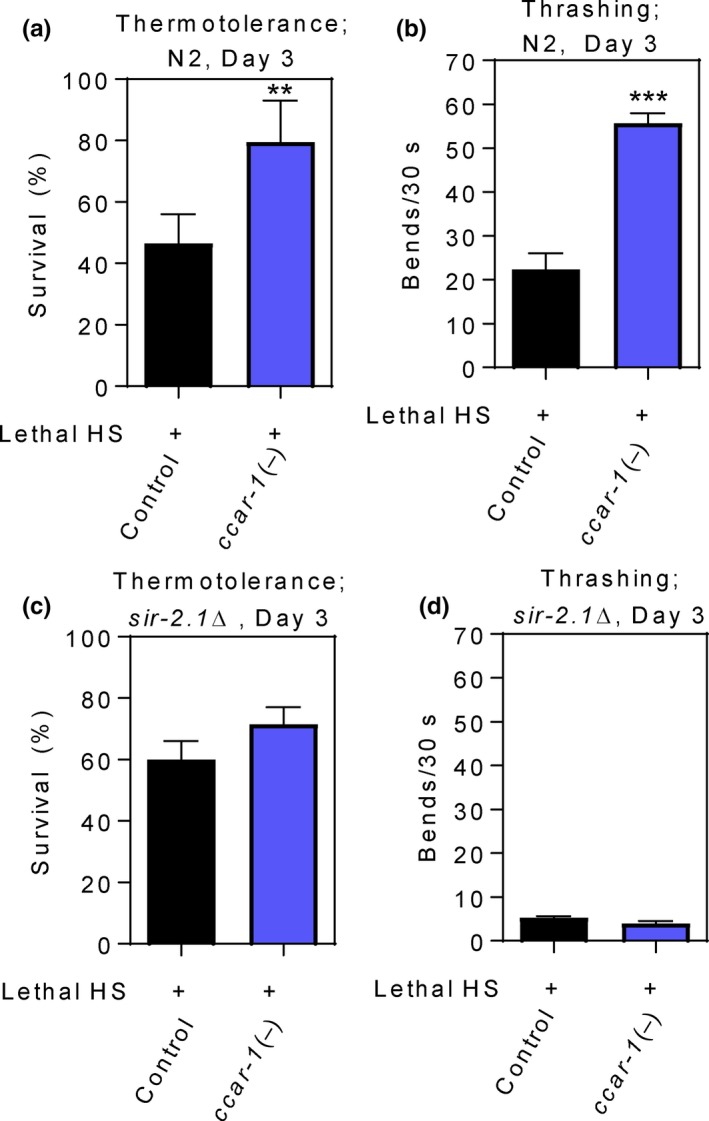
*ccar‐1* RNAi promotes thermotolerance and thrashing in aging worms in a *sir‐2.1*‐dependent manner. (a) Thermotolerance was measured in wild‐type (N2) worms fed control RNAi or *ccar‐1* RNAi from the L1 larval stage until day 3 of adulthood prior to treatment with a lethal (50% survival in the control) 42°C 1‐hr HS followed by a 12‐hr recovery. (b) Thrashing was measured as number of body bends/30 s for survivors of the lethal HS in (a). (c) Thermotolerance was measured in *sir2.1Δ* worms fed control RNAi or *ccar‐1* RNAi from the L1 larval stage until day 3 of adulthood prior to treatment with a lethal 42°C 1‐hr HS followed by a 12‐hr recovery. (d) Thrashing was measured in number of body bends/30 s for survivors of the lethal HS in (c). For (a‐d), significance was determined using the Bonferroni post hoc test, where **p < 0.01, ***p < 0.001

To examine the impact of CCAR‐1 on stress resistance and motility, we next assessed survival upon exposure to a lethal HS and thrashing in aging worms in response to *ccar‐1* RNAi (Figure 4). Wild‐type (N2) or *sir‐2.1Δ* worms were fed control RNAi or *ccar‐1* RNAi from the L1 larval stage until day 3 of adulthood, treated with a lethal 42°C 1‐hr HS, and survivors were scored 12 hr after the HS. This HS treatment condition resulted in ~50% survival in wild‐type worms fed control RNAi, and ~80% survival in wild‐type worms fed *ccar‐1* RNAi (Figure 4a). *ccar‐1* RNAi not only promoted survival during a lethal HS, but also enhanced the motility of worms that survived the lethal HS which was measured in number of body bends/30 s (Figure 4b). Wild‐type worms fed control RNAi that survived a lethal HS moved at a rate of 20 body bends/30 s, whereas worms fed *ccar‐1* RNAi moved at a rate of 55 body bends/30 s. Under non‐HS conditions, *ccar‐1* RNAi provided only a slight increase in thrashing activity (Supporting Information Figure S4), suggesting that the enhanced motility effect is dependent, at least in part, on HSF‐1. Unexpectedly, the *sir‐2.1Δ* worms had a higher rate of thermotolerance than the N2 worms (Figure 4c). However, the ability of *ccar‐1* RNAi to significantly enhance thermotolerance was no longer observed upon *sir‐2.1* deletion (Figure 4c). SIR‐2.1 therefore may have complex functions in regulating thermotolerance. Additionally, *sir‐2.1Δ* worms that survived the severe HS exhibited a dramatic decrease in motility regardless of *ccar‐1* RNAi treatment (Figure 4d). These data therefore suggest that CCAR‐1 would normally reduce stress resistance and motility through inhibition of SIR‐2.1.

### 2.5 *ccar‐1* RNAi promotes longevity in a SIR‐2.1‐dependent manner

**Figure 5:**
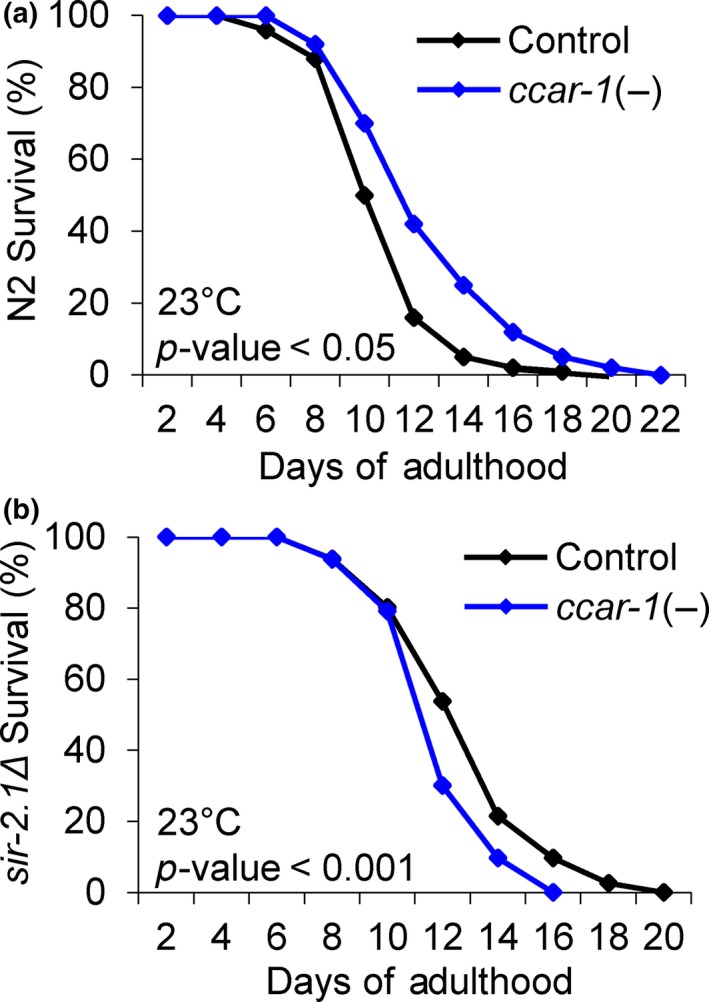
*ccar‐1* RNAi increases longevity in a *sir‐2.1*‐dependent manner. (a) Lifespan analysis was performed at 23°C in wild‐type (N2) worms fed control RNAi or *ccar‐1* RNAi throughout lifespan. (b) Lifespan analysis was performed at 23°C in *sir2.1Δ* worms fed control RNAi or *ccar‐1* RNAi throughout lifespan. For (a–b), worms were scored every other day for survival, and significance was determined using the Mantle–Cox rank test

Next, we performed lifespan assays to assess the impact of CCAR‐1 on longevity (Figure 5). Wild‐type (N2) or *sir‐2.1Δ* worms were fed control RNAi or *ccar‐1* RNAi from the L1 larval stage throughout life. Worms were scored every other day starting at day 1 of adulthood for survival and scored as dead when nonresponsive to poking with a platinum wire. Wild‐type worms fed control RNAi had a median survival of 10 days, and a maximum survival of 18 days, whereas worms fed *ccar‐1* RNAi had a median survival of 12 days, and a maximum survival of 22 days (Figure 5a, Supporting Information Figure S5). Unexpectedly, *sir‐2.1Δ* worms showed a slightly longer lifespan than N2 worms. However, the increase in median and maximum lifespan observed in response to treatment with *ccar‐1* RNAi was dependent on SIR‐2.1, as *sir‐2.1Δ* worms show a 4‐day decrease in longevity when fed *ccar‐1* RNAi as compared to the RNAi control (Figure 5b). Thus, CCAR‐1 would normally decrease longevity via inhibition of SIR‐2.1.

### 2.6 *ccar‐1* RNAi promotes proteostasis in a *C. elegans* Huntington's disease model

**Figure 6:**
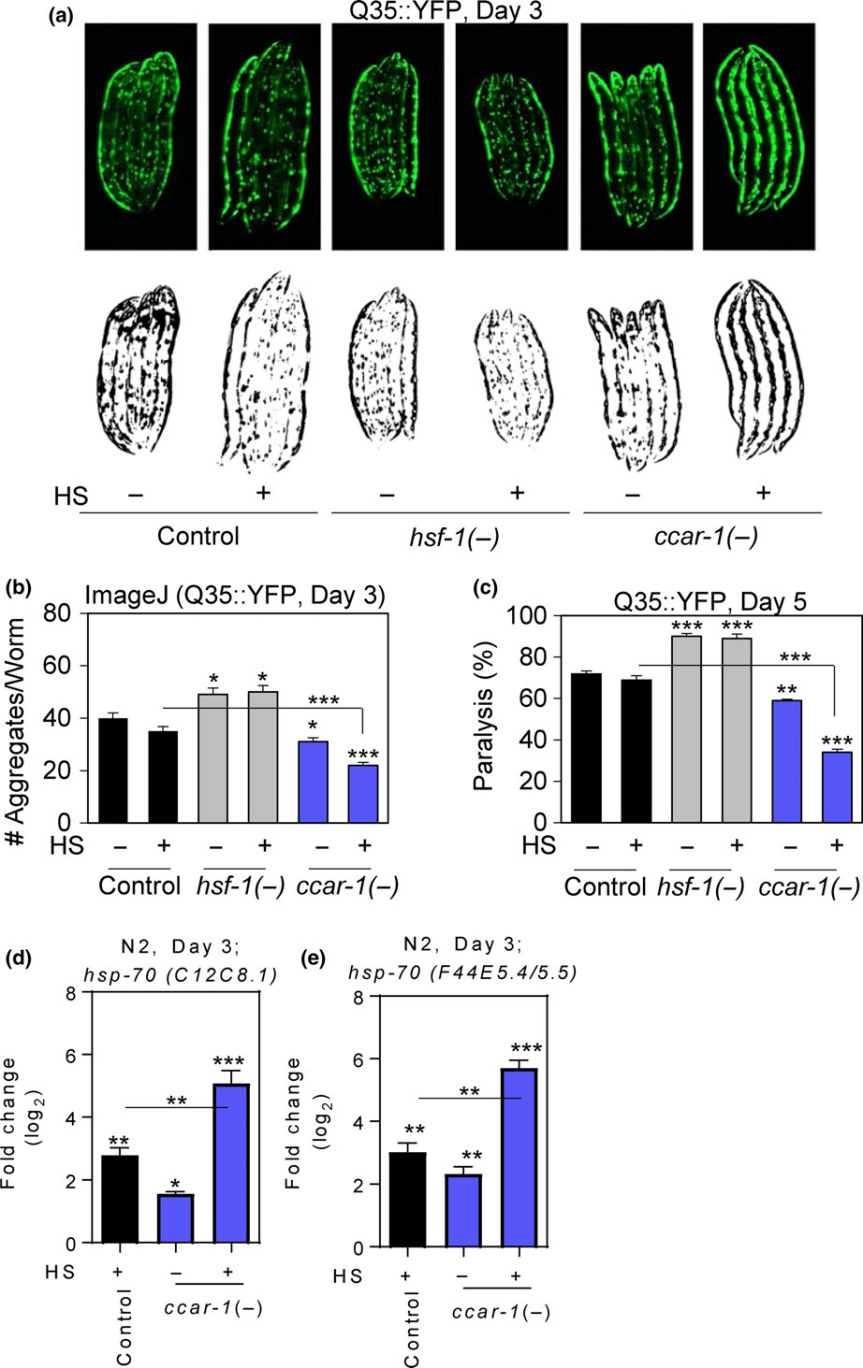
*ccar‐1* RNAi decreases polyglutamine aggregation and paralysis in a Huntington's disease model and inhibits the age‐related decline of the HSR. (a) Fluorescent images of a *C. elegans* Huntington's disease model containing 35 polyglutamine repeats fused to YFP under the control of a muscle‐specific promoter (Q35::YFP) were fed control RNAi, *hsf‐1* RNAi, or *ccar‐1* RNAi from the L1 larval stage until day 3 of adulthood prior to treatment with or without a 15‐min HS followed by a 12‐hr recovery. Threshold‐adjusted images are shown below the fluorescent images. (b) ImageJ was used to quantify the number of polyglutamine aggregates/worm using the threshold‐adjusted images from A for 50 worms/condition in biological triplicates. (c) Paralysis was measured in Q35::YFP worms that were fed control RNAi, *hsf‐1* RNAi, or *ccar‐1* RNAi from the L1 larval stage until day 5 of adulthood prior to treatment with or without a 15‐min HS followed by a 12‐hr recovery. (d‐e) qRT–PCR was used to measure the expression of the *hsp‐70* family members *C12C8.1* and *F44E5.5*/*F44E5.4* in wild‐type worms given the same treatment conditions as in (a). For (b–e), significance was determined using the Bonferroni post hoc test, where *p<0.05, **p < 0.01, ***p < 0.001

To observe the impact of CCAR‐1 on proteostasis, we used a *C. elegans* Huntington's disease model to observe polyglutamine aggregate formation and toxicity in response to *ccar‐1* RNAi (Figure 6). The Huntington's disease model used here (strain AM140) contains 35 polyglutamine repeats fused to YFP (Q35::YFP) under the control of a muscle‐specific promoter and develops insoluble protein aggregates in an age‐dependent manner in the body wall muscle (Morley, Brignull, Weyers, & Morimoto, 2002). Synchronous Q35::YFP worms were fed control RNAi, *hsf‐1* RNAi, or *ccar‐1* RNAi from the L1 larval stage until day 3 of adulthood prior to treatment with or without a 15‐min HS, followed by a 12‐hr recovery. Fluorescent images, as well as threshold‐adjusted images, are shown (Figure 6a). ImageJ was used on the threshold‐adjusted images to quantify the number of aggregates per worm for each treatment condition (Figure 6b). As expected, *hsf‐1* RNAi increased aggregate formation. In the absence of HS, *hsf‐1* RNAi lead to an increase of 10 aggregates/worm, and in the presence of HS, *hsf‐1* RNAi leads to an increase of 12 aggregates/worm, as compared to the respective controls. Interestingly, *ccar‐1* RNAi decreased aggregate formation by 9 aggregates/worm in the absence of HS and by 12 aggregates/worm during HS, as compared to the respective controls. CCAR‐1 therefore normally decreases proteostasis in a *C. elegans* Huntington's disease model.

We next examined paralysis in the Huntington's disease model in order to assess the toxicity associated with aggregate formation (Figure 6c). Synchronous Q35::YFP worms were fed control RNAi, *hsf‐1* RNAi, or *ccar‐1* RNAi from the L1 larval stage until day 5 of adulthood prior to treatment with or without a 15‐min HS, followed by a 12‐hr recovery. As expected, treatment with *hsf‐1* RNAi increased the number of paralyzed worms. This increase was by 19% and was not enhanced by HS. Treatment with *ccar‐1* RNAi decreased the number of paralyzed worms by 12% in the absence of HS and by 25% in the presence of HS. These data suggest that CCAR‐1 normally antagonizes proteostasis and leads to increased aggregate‐associated toxicity.

To determine whether the decrease observed in aggregate formation in response to *ccar‐1* RNAi may be due to increased *hsp‐70* mRNA levels in day 3 worms, we performed qPCR on *C. elegans* fed *ccar‐1* RNAi (Figure 6d–f). Consistent with the impaired HSR in aged worms (Labbadia & Morimoto, 2015), the induction of *hsp‐70* mRNA by HS is lower in day 3 worms than in L4 worms (compare Figure 6d–f with Figure 3). Interestingly, treatment with *ccar‐1* RNAi increased the expression of each *hsp‐70* family member in the absence of HS by 1.8‐ to 2‐fold at day 3, while further enhancing *hsp‐70* expression when combined with HS. *ccar‐1* RNAi therefore helps to prevent age‐associated decline of the HSR and is beneficial for maintaining proteostasis during aging. These results suggest that CCAR‐1 normally dampens the HSR with age.

## 3 DISCUSSION

The study of CCAR‐1 in *C. elegans* is gaining interest. CCAR‐1/LST‐3 was first described as being involved in a lateral signaling pathway that regulates vulval development (Yoo, Bais, & Greenwald, 2004). *ccar‐1* was then identified via an RNAi screen to contribute to the maintenance of a functional hemidesmosome (Zahreddine, Zhang, Diogon, Nagamatsu, & Labouesse, 2010), and recent findings suggest that CCAR‐1 affects alternative splicing of the *unc‐52* gene during hemidesmosome formation (Fu et al., 2018). CCAR‐1 is localized to the nuclei of most cells at different developmental stages (Fu et al., 2018).

In this study, we have identified CCAR‐1, a CCAR2 ortholog, as a negative regulator of the HSR in *C. elegans*. We have found that CCAR‐1 modulates *hsp‐70* promoter activity (Figure 1), HSF‐1 acetylation (Figure 2a,b), and HSF‐1‐binding to the *hsp‐70* promoter during HS (Figure 2c,d). We note that while CCAR‐1 decreases total HSF‐1 acetylation levels both with and without HS (Figure 2a,b), the effects on total HSF‐1 acetylation changes are modest in comparison to the effects on *hsp‐70* promoter activity (Figure 1a,b) and on DNA‐binding activity (Figure 1c,d). mRNA expression does not appear to be regulated by HS or by HSF‐1 as determined by RNA sequencing experiments (Brunquell et al., 2016). Future work will test whether there are certain acetylation sites within *C. elegans* HSF‐1 with specific functions that may allow for this discrepancy, or whether CCAR‐1 causes other effects in addition to changing HSF‐1 acetylation that may alter the heat‐shock response.

As mammalian DBC1 is known to inhibit SIRT1, we sought to see whether alterations in physiological readouts of the HSR by CCAR‐1 were dependent on SIR‐2.1. We found that worms treated with *ccar‐1* RNAi show an increase in HS‐inducible *hsp‐70* gene expression that is dependent on SIR‐2.1. Additionally, SIR‐2.1 is required for *ccar‐1* RNAi to promote stress resistance, thermotolerance, motility, longevity, and proteostasis, while also preventing an age‐dependent decline in the HSR. We predict that CCAR‐1 inhibits the deacetylase activity of SIR‐2.1, thus allowing more HSF‐1 to exist in an acetylated state that may not bind to DNA. Future analyses of HSF‐1 acetylation and DNA binding in a *sir‐2.1* mutant strain will be required to validate this prediction. Enhancing the HSR by modulating sirtuin activity is one strategy that may be utilized to promote proteostasis and longevity. Based on our work, we can now add a predicted function for the CCAR‐1 protein in negatively regulating the HSR.

Modulating the HSR by controlling SIRT1 activity is a promising new method of promoting proteostasis. The likelihood of developing diseases of protein dysfunction, such as neurodegenerative disorders, is increased upon aging, due in part to the decline of the HSR during the aging process (Ben‐Zvi, Miller, & Morimoto, 2009; Labbadia & Morimoto, 2015). Activators of the HSR have been suggested as possible therapeutic strategies for diseases of aging (Balch, Morimoto, Dillin, & Kelly, 2008; Calamini & Morimoto, 2012; Neef, Turski, & Thiele, 2010; Westerheide & Morimoto, 2005), but many of the small molecules known to modulate HSF1 activity have cytotoxicity and poor bioavailability. Our data suggest that modulating SIR‐2.1 activity may prevent an age‐associated decline in the HSR and may prevent polyglutamine aggregation in a *C. elegans* Huntington's disease model. Therefore, our studies support the use of sirtuin modulators for diseases of protein quality control to promote healthy aging.

## 4 EXPERIMENTAL PROCEDURES

### 4.1 *C. elegans* strains and growth conditions

The following *C. elegans* strains were used in this study: Bristol N2 (wild‐type), *sir‐2.1Δ* (LG339) (Viswanathan & Tissenbaum, 2013; Viswanathan, Kim, Berdichevsky, & Guarente, 2005), *sir‐2.3Δ* (RB654) (Barstead & Moerman, 2006), Q35::YFP (AM140) (38), HSF‐1::GFP (EQ73) (Chiang et al., 2012), and the *pC12C8.1::GFP* reporter strain (39). All strains were grown at 23°C and maintained on standard nematode growth media (NGM) containing the *Escherichia coli* strain OP50. Age synchronization was accomplished by hypochlorite treatment.

### 4.2 RNA interference

Synchronous L1 nematodes were placed onto standard NGM plates supplemented with 25 µg/ml ampicillin and 1 mM isopropyl‐beta‐ᴅ‐thiogalactopyranoside and seeded with either HT115 bacteria containing an empty vector (L4440, control), or with sequence‐verified gene‐specific RNAi strains isolated from the Ahringer RNAi library (40).

### 4.3 HS treatment

*Caenorhabditis elegans* were grown on RNAi plates as indicated, wrapped in parafilm and then submerged in a 33°C water bath for the allotted times. Prior to RNA extraction, animals were recovered for 15 min at growth temperature. Prior to GFP analysis, animals were recovered for 6 hr at growth temperature.

### 4.4 EX‐527 compound treatment

EX‐527 (Sigma, cat#E7034) was diluted in DMSO and added to NGM after autoclaving at a final concentration of 1 µM. Synchronous worms were grown on vehicle control or EX‐527 supplemented plates from the L1 larval stage to the L4 larval stage prior to analyses.

### 4.5 Fluorescence microscopy

Animals were anesthetized with 10 mM levamisole and photographed using an EVOS fluorescence microscope. Image processing was accomplished using Adobe Photoshop© (Adobe Systems Incorporated). Quantification of fluorescence intensity was performed using ImageJ Software (v. 1.44; https://imagej.nih.gov/ij/).

### 4.6 Immunoblotting

Animals were harvested in Buffer C (20 mM HEPES pH 7.9, 25% glycerol, 0.42 M NaCl, 1.5 mM MgCl2, 0.2 mM EDT, and 0.5 mM DTT) with the addition of Halt™ protease inhibitors (Pierce, cat# 78,430). Protein was extracted by sonication with a Diagenode Bioruptor. Antibodies used were an anti‐GFP antibody (Abcam, cat# ab290) and an anti‐actin antibody (Amersham, cat#JLA20‐C). Quantification of band intensity was performed using ImageJ Software (v. 1.44; https://imagej.nih.gov/ij/).

### 4.7 Quantitative RT–PCR

RNA was extracted with TRIzol® reagent (Ambion®, cat# 15,596–026) by standard protocol. RNA was reverse‐transcribed using a High Capacity cDNA Reverse Transcription Kit (Applied Biosystems, cat# 4,368,814). cDNA was diluted to 50 ng/µl to be used as a template for qRT–PCR performed with the StepOne Plus Real‐time PCR system (Applied Biosystems, cat # 4,376,600) using iTaq™ Universal SYBR® Green Supermix (Bio‐Rad, cat# 1,725,121) according to the manufacturer's instructions. Results show averages of independent biological triplicates performed in technical duplicates. Statistical analysis was performed with GraphPad (GraphPad Software, http://www.graphpad.com) using ANOVA followed by the Bonferroni posttest. Primer sequences are supplied (Supporting Information Table S1).

### 4.8 Lifespan analysis

All lifespan assays were performed at 23°C with about 100 worms per condition in biological triplicate. Animals were transferred to fresh plates daily for 5 days to avoid progeny contamination. Adult worms were scored every other day and counted as dead when no response was observed by poking with a platinum wire. Survivability was plotted using GraphPad Prism v.6 (GraphPad Software, http://www.graphpad.com), and statistical analysis was done using the Kaplan–Meier log‐rank test.

### 4.9 Thermotolerance and thrashing assay

Hundred synchronized L1 nematodes were grown on control (L4440) or gene‐specific RNAi plates at growth temperature (23°C), transferred to new plates daily until day 3 of adulthood, and then submerged in a 42°C water bath for 1 hr which allowed for a 50% survival rate. Animals were scored 12 hr later and marked as dead when nonresponsive to poking by a platinum wire. Live animals were then scored for motility by assessing body bends when placed into a drop of M9 on a glass slide. After acclimation to the M9 for 10 s, body bends were counted for 30 s.

### 4.10 Protein aggregation assay

Q35::YFP nematodes were synchronized and grown on empty vector (L4440, control) or gene‐selected RNAi plates. Worms were picked to fresh plates daily after first progeny development until day 3 of adulthood, and then, plates were submerged in a 33°C water bath for 15 min and allowed to recover for 12 hr at growth temperature. Protein aggregates were scored in a blind analysis of at least 50 worms per condition in independent biological triplicates using ImageJ analysis as previously described (Brunquell, Bowers, & Westerheide, 2014).

### 4.11 Paralysis assay

Q35::YFP nematodes were synchronized and grown on empty vector (L4440, control) or gene‐selected RNAi plates. Worms were picked to fresh plates daily after first progeny development until day 5 of adulthood, and then, plates were submerged in a 33°C water bath for 15 min and allowed to recover for 12 hr at growth temperature. Paralysis was determined by transferring live worms to a corresponding RNAi plate and observing movement within a 2‐min period. Worms that did not move within the timeframe were considered paralyzed.

### 4.12 Acetylation assay

Approximately 13,000 HSF‐1::GFP (EQ73) worms were bleach‐synchronized and placed onto gene‐specific RNAi plates until reaching the L4 stage prior to being left untreated or treated with heat shock as described above. Worms were collected in HLB Buffer [50 mM HEPES‐KOH, pH 7.5, 150 mM NaCl, 1 mM EDTA, 0.1% (wt/vol) sodium deoxycholate, 1% (vol/vol) Triton X‐100, 0.1% (wt/vol) SDS, Halt™ protease inhibitors (Pierce, cat# 78,430), 1 μM trichostatin A, 1 μM nicotinamide, and 1 μM EX‐527] and homogenized with a Dounce homogenizer prior to centrifugation at 14,000× *g* for 20 min at 4°C. Protein was quantified by Bradford assay, and immunoprecipitation was accomplished using 1 mg protein extract and an anti‐GFP polyclonal antibody (Abcam, cat# ab290). The antibody–protein complex was allowed to form overnight at 4°C with rotation. 50 μl of salmon sperm DNA/protein‐A agarose beads (Millipore, cat# 16–157) was then added and allowed to incubate for 1 hr at 4°C. The beads were washed 3 times with HLB buffer supplemented with 1 μM trichostatin A, 1 μM nicotinamide, and 1 μM EX‐527 before being boiled in Laemmli buffer. The resulting supernatant was then resolved on a 10% SDS‐PAGE gel and transferred to a PVDF membrane. The blot was incubated with anti‐GFP antibody (Abcam, cat# ab290) and with anti‐AcK antibody (Cell Signaling #9,441).

### 4.13 Chromatin immunoprecipitation procedure and data analysis

Chromatin immunoprecipitation (ChIP) was performed essentially as previously described (Mukhopadhyay, Deplancke, Walhout, & Tissenbaum, 2008). Approximately 13,000 HSF‐1::GFP (EQ73) worms were bleach‐synchronized and placed onto gene‐specific RNAi plates until reaching the L4 stage prior to being left untreated or given a 15‐min HS as described above. Worms were collected, cross‐linked with 1% formaldehyde, lysed with a homogenizer, and quenched with glycine before being sonicated with a Diagenode Bioruptor for 10 min with 30‐s pulses. Protein was quantified and technical triplicates were performed with 2 mg of total protein diluted in HLB buffer (50 mM HEPES‐KOH, pH 7.5, 150 mM NaCl, 1 mM EDTA, 0.1% [wt/vol] sodium deoxycholate, 1% [vol/vol] Triton X‐100, 0.1% [wt/vol] SDS, and Halt™ protease inhibitors [Pierce, cat# 78430]). 1% of each sample was saved as the input. An anti‐GFP polyclonal antibody (Abcam, cat# ab290) and the IgG antibody were used. The antibody–protein complex was allowed to form overnight at 4°C. 50 μL of salmon sperm DNA/protein‐A agarose beads (Millipore, cat# 16–157) was added to the diluted supernatant and allowed to incubate for 1 hr at 4°C. The antibody–protein–agarose bead complex was washed 2 times with WB1 (50 mM HEPES‐KOH, pH 7.5, 150 mM NaCl, 1 mM EDTA pH 8.0, 1% sodium deoxycholate, 1% Triton X‐100, 0.1% SDS and HALT protease inhibitors), WB2 (50 mM HEPES‐KOH, pH 7.5, 1 M NaCl, 1 mM EDTA, pH 8.0, 0.1% sodium deoxycholate, 1% Triton X‐100, 0.1% SDS and HALT protease inhibitors), WB3 (50 mM Tris–Cl, pH 8.0, 0.25 mM LiCl, 1 mM EDTA, 0.5% NP‐40 and 0.5% sodium deoxycholate) and then with 1xTE. The ChIP samples and the input samples were placed at 45°C for 2 hr with the addition of proteinase K buffer/proteinase K. The samples were then reverse cross‐linked with an overnight incubation at 65°C, and DNA was purified using a PCR purification kit. qRT–PCR was performed using primers flanking a HS element in the promoter of the *hsp‐70* (*C12C8.1*) gene or using upstream primers. Percent input was calculated by first adjusting the raw Ct values of the diluted input to 100% by subtracting 6.644 (log_2_ of the dilution factor). The square of the average Ct values of the ChIP samples, subtracted from the adjusted input, was then multiplied by 100 to obtain the percent input.

### 4.14 Statistical analyses

Statistical analyses were carried out with GraphPad Software (GraphPad Software, La Jolla, CA, USA, https://www.graphpad.com). All error bars are representative of standard deviation between independent biological replicates, as indicated.

## CONFLICT OF INTEREST

None declared.

## AUTHOR CONTRIBUTIONS

JB, RR, and SW designed the study. JB and PB performed the experiments in Figures 1 and 3. JB, SM, PB, and AS performed the experiments in Figures 2, 4, 5, and 6. AD performed the experiment in Supporting Information Figure S1 and DL performed the experiment in Supporting Information Figure S4. JB performed data analyses. JB and SW wrote the manuscript.
