# Supplementary figures and images for: CCAR‐1 is a negative regulator of the heat‐shock response in Caenorhabditis elegans
Source: Aging Cell. 2018 Jul 12;17(5):e12813. doi: 10.1111/acel.12813 (PMC6156500; doi:10.1111/acel.12813)

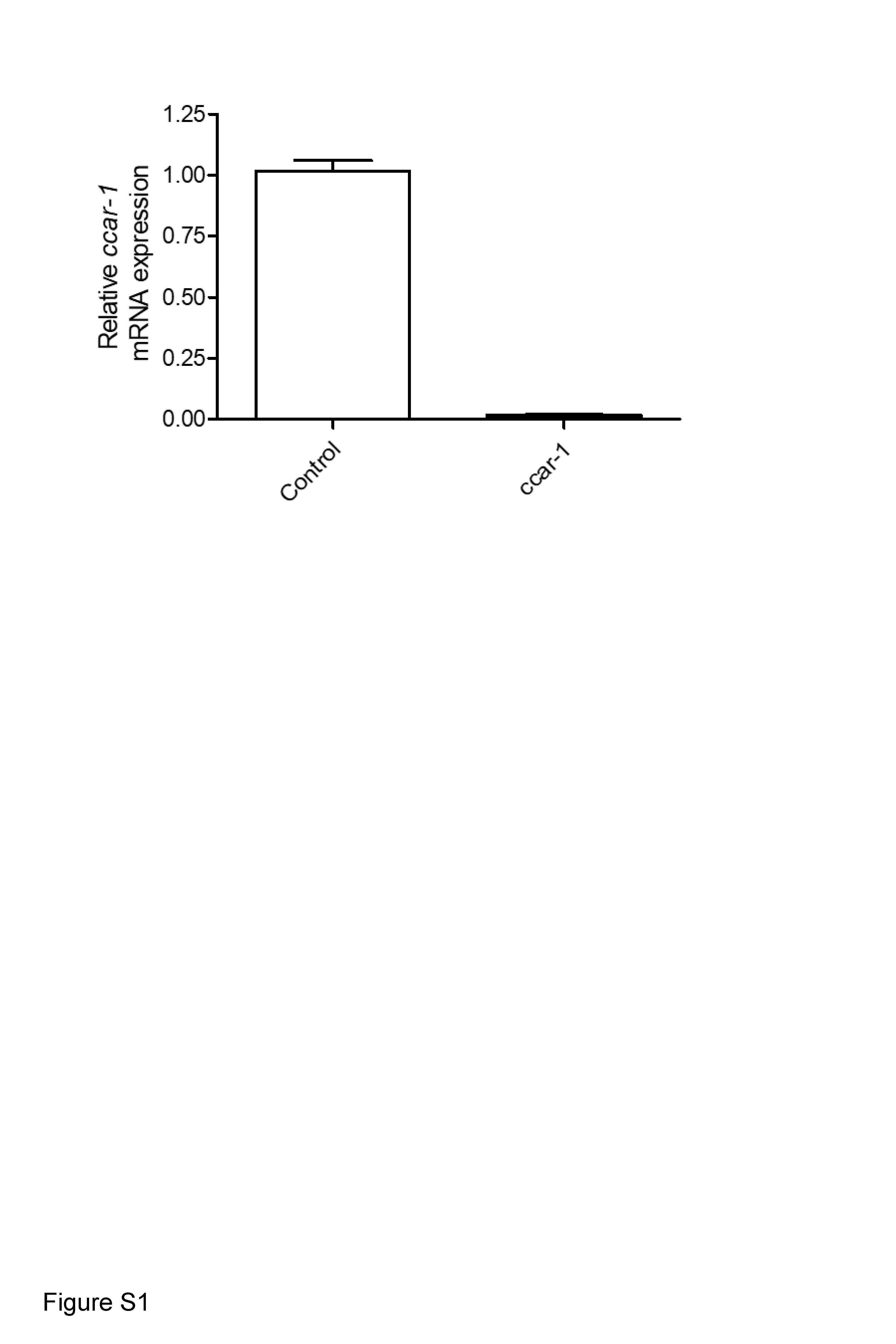

Supplement: Supplementary file 1 [file ACEL-17-e12813-s001.tif]

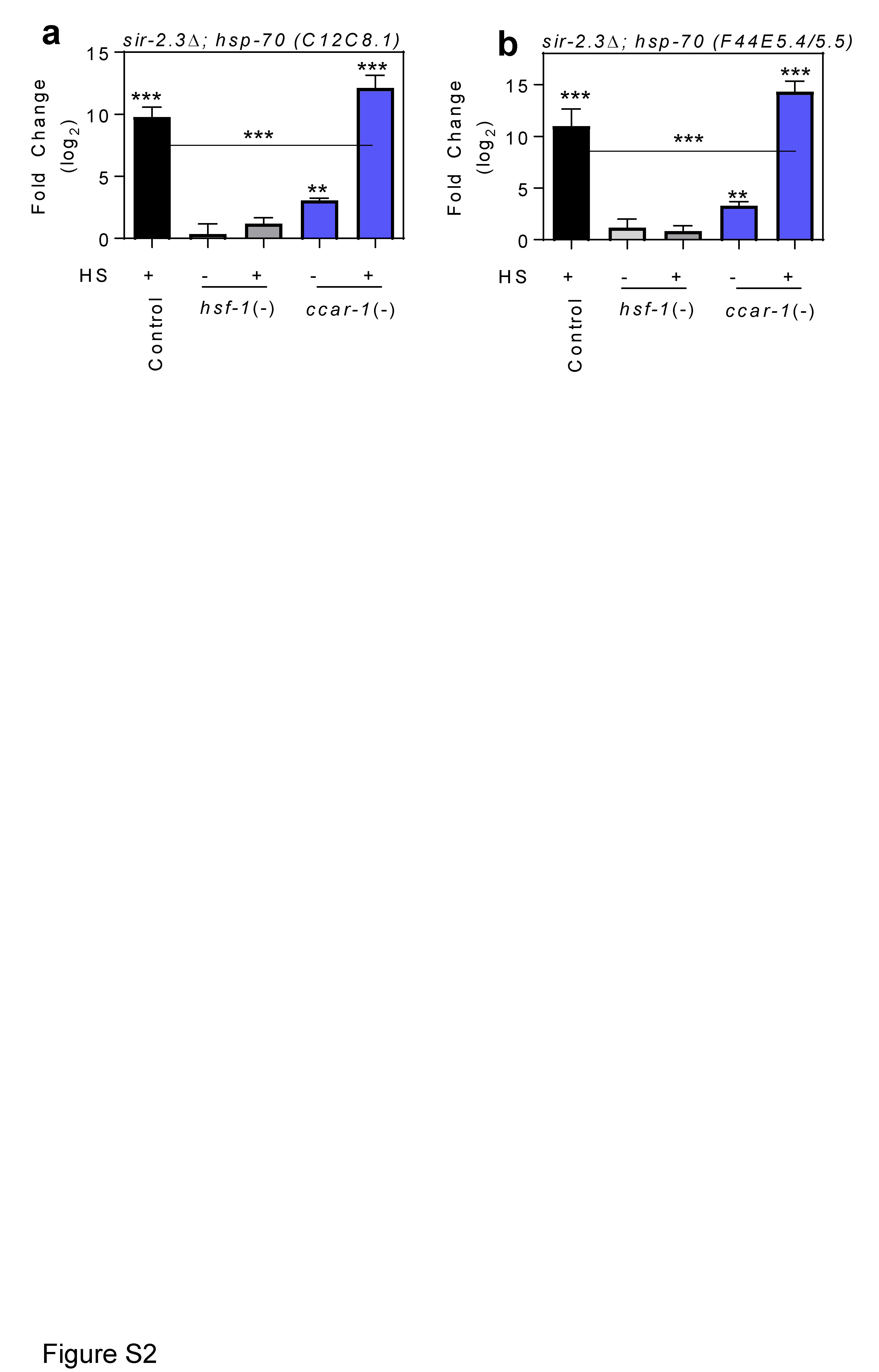

Supplement: Supplementary file 2 [file ACEL-17-e12813-s002.tif]

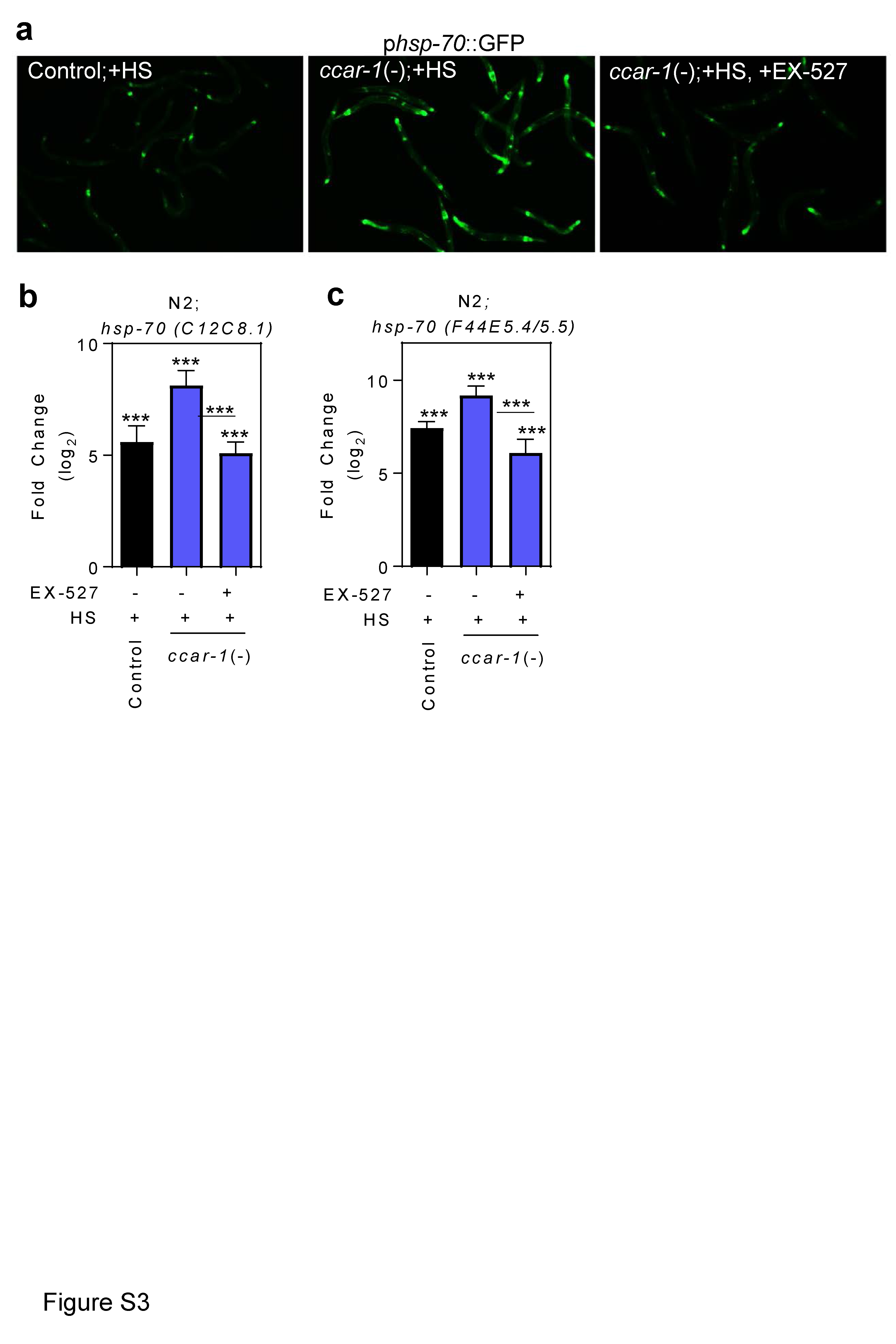

Supplement: Supplementary file 3 [file ACEL-17-e12813-s003.tif]

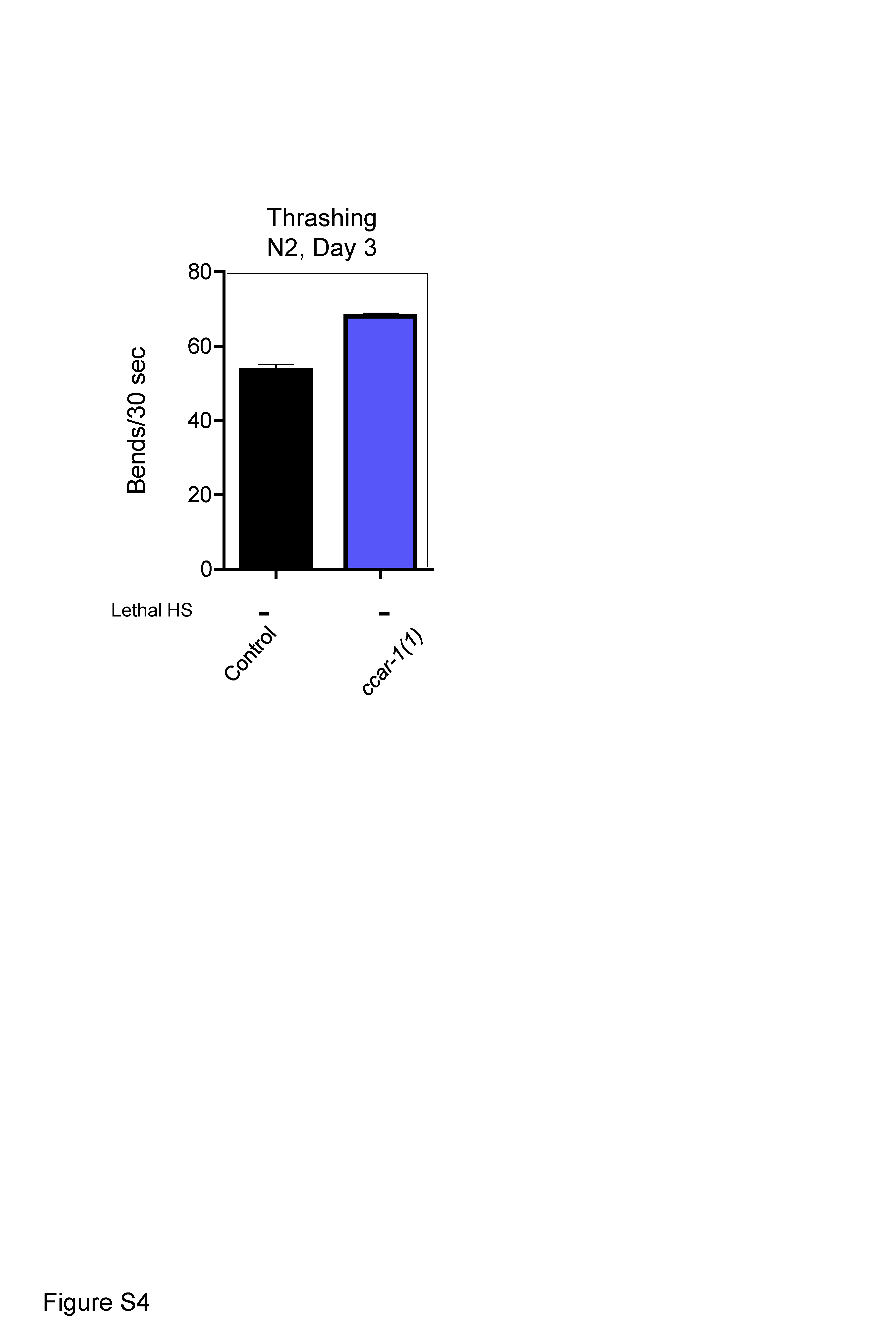

Supplement: Supplementary file 4 [file ACEL-17-e12813-s004.tif]

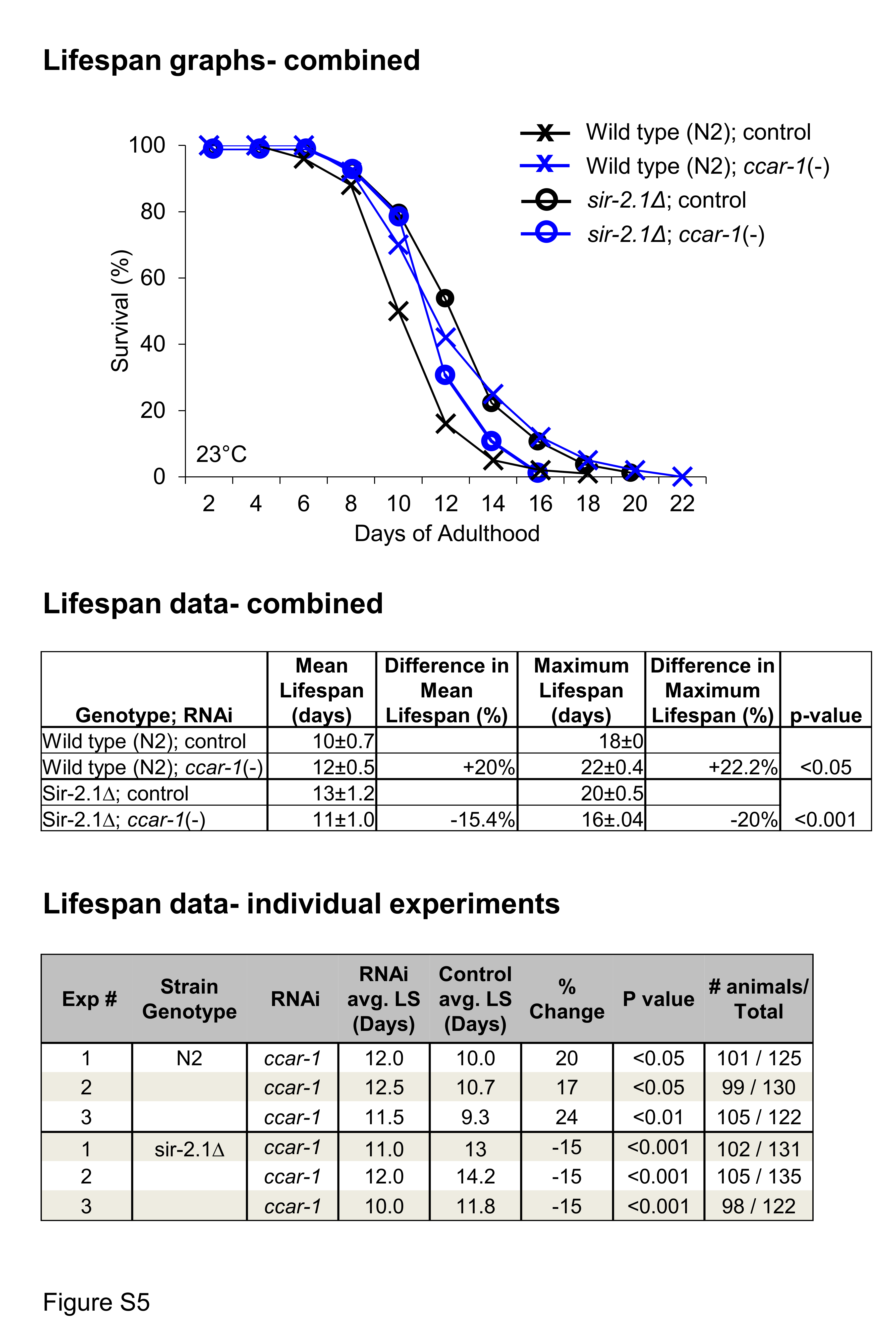

Supplement: Supplementary file 5 [file ACEL-17-e12813-s005.tif]

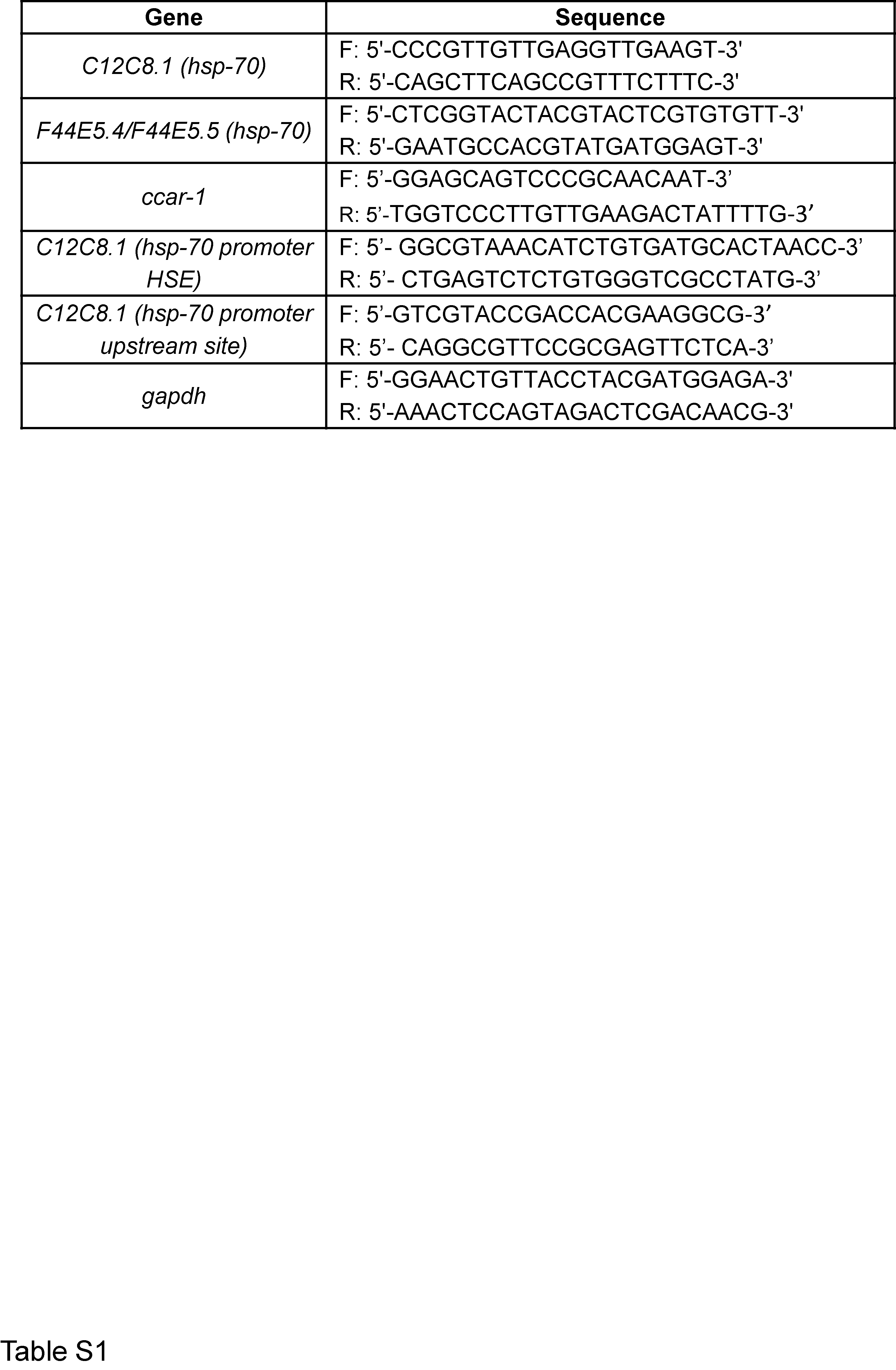

Supplement: Supplementary file 6 [file ACEL-17-e12813-s006.tif]
